# Influence of the Activation of NMDA Receptors on the Resting Membrane Potential of the Postsynaptic Cell at the Neuromuscular Junction

**Published:** 2018

**Authors:** S. E. Proskurina, K. A. Petrov, E. E. Nikolsky

**Affiliations:** Open Laboratory of Neuropharmacology, Kazan Federal University, Kremlyovskaya Str., 18, Kazan, 420008, Russia; Laboratory of Biophysics of Synaptic Processes, Kazan Institute of Biochemistry and Biophysics, Russian Academy of Sciences, Lobachevsky Str., 2/31, Kazan, 420111, Russia; A.E. Arbuzov Institute of Organic and Physical Chemistry, Russian Academy of Sciences, Arbuzov Str., 8, Kazan, 420029, Russia; Department of Medical and Biological Physics, Kazan State Medical University, Butlerova Str., 49, Kazan, 420012, Russia

**Keywords:** Neuromuscular transmission, NMDA receptor, glutamate, glycine, electrophysiology

## Abstract

Impaired function or insufficient expression of glutamate N-methyl-D-aspartate (NMDA) receptors underlies a number of brain pathologies; these receptors are, therefore, regarded as a pharmacological target for many neuroactive drugs. It was shown that in the CNS, this type of glutamate receptors participate in the processes of neuronal excitation, synaptic plasticity [1, 2], and excitotoxicity in neurodegenerative diseases and are also involved in the pathogenesis of epilepsy and seizures. However, until recently, the presence and activity of NMDA receptors beyond the CNS had never been considered. This research shows that activation of NMDA receptors at the mammalian neuromuscular junction alters the resting membrane potential of the postsynaptic cell evoked by cation entry through the receptor-associated channel.

## INTRODUCTION

Neuromuscular synaptic transmission is indispensable for the process of human life, as it is the mechanism that transfers cerebral commands to activate muscle contractions. The neuromuscular junction (NMJ) is a synapse composed of the presynaptic motor nerve terminal, the synaptic cleft, and the postsynaptic region of muscle fiber. The NMJ is a chemical-type synapse in which transmission of basic signals is mediated by acetylcholine (ACh). However, it is worth mentioning that other neurotransmitters (glutamate, ATP, GABA) have been shown to exist in this presumably cholinergic synapse, whose putative role is fine-tuning of ACh release [3, 4, 5].

NMDA receptors (NMDAR) are ionotropic ligand-gated receptors associated with the cation-permeable channel [6]. Simultaneous presence of two co-agonists (glutamate and glycine) and removal of the magnesium block are required to activate them [7]. It has been shown that Mg^2+^ blockade can be voided by membrane depolarization under native conditions or by using a Mg^2+^-free Ringer solution, in experiments.

We have previously shown the role played by these receptors in the modulation of ACh release [8] and regulation of acetylcholinesterase activity [9] at mammalian NMJ. Furthermore, postsynaptic localization of the NMDA receptor NR1-subunit has been demonstrated [10]. Such localization suggests that the agonists of these receptors may change membrane excitability (namely, the resting membrane potential) with the development of depolarization.

## MATERIALS AND METHODS

Male Wistar rats (200–300 g body weight) were used for all the experiments. The experiments were carried out in compliance with the guidelines for using labora tory animals of the Kazan Federal University and Kazan Medical University (ethical approval by the Institutional Animal Care and Use Committee of the Kazan State Medical University N9-2013). The experimental protocol met the requirements of the European Communities Council Directive 86/609/EEC and was approved by the Ethics Committee of the Kazan Medical University. All possible efforts were made to minimize animal suffering and to reduce the number of animals used. The experiments were performed using isolated nerve–muscle preparations of the EDL (extensor digitorum longus) muscle excised from ether-anaesthetized rats. Isolated muscles with a nerve stump (10–15 mm long) were placed in a chamber and superfused (at a rate of 2–3 mL/min) with an oxygenated Ringer–Krebs rat solution with the following composition (mM): 120.0 NaCl, 5.0 KCl, 2.0 CaCl_2_, 1.0 MgCl_2_, 23.0 NaHCO_3_, 1.0 NaH_2_PO_4_, and 11.0 glucose; or Mg^2+^-free solution: 121.0 NaCl, 5.0 KCl, 2.0 CaCl_2_, 0.0 MgCl_2_, 23.0 NaHCO_3_, 1.0 NaH_2_PO_4_, and 11.0 glucose. The pH was maintained at 7.2–7.4.

Changes in the membrane potential were recorded at the endplate region of the muscle fibers using the standard current clamp technique at 20–22 °C as described by Petrov et al. [11]. Glass microelectrodes (resistance, 10–15 MΩ) were filled with 3 M KCl.

Glutamate, glycine, AP5, and 5,7-DCKA were purchased from Sigma-Aldrich (USA); μ-conotoxin was purchased from Alamone Lab (Israel). The animals received the agents through a perfusion system.

The statistical significance of the results was assessed using the unpaired Student’s t-test; the difference between two data sets was considered significant at *p * < 0.05; errors are shown as a standard deviation (SD).

## RESULTS AND DISCUSSION

It was suggested that, in the presence of functionally active NMDA receptors on the postsynaptic membrane, opening of receptor-associated channels caused by the application of agonists results in the entry of cations, leading to membrane depolarization. The depolarization intensity would depend on the quantity of activated receptors and concentration of extracellular ions. However, it was demonstrated that, at the resting membrane potential (RMP), Mg^2+^ blocks the NMDAR channel but these ions could be dislodged by depolarization or, in the experiments, by usage of a magnesium-free solution [12, 13].

Application of glutamate (100 μM) and NMDAR co-agonist glycine (700 μM) in a Mg^2+^-free solution reduced the resting membrane potential by 6.5% (74.2 ± 0.3 mV, *n *= 140 vs. 79.4 ± 0.2 mV in control, *n *= 270, *p * < 0.05, *Figure*). In order to elucidate whether this effect was due to the activation of NMDARs, we performed the experiments using APV (DL-2-amino-5-phosphonopenthatoic acid), a selective reversible NMDAR blocker. Addition of 500 μM APV had no effect on the RMP; subsequent application of glutamate and glycine in the presence of the blocker evoked a smaller depolarization amounting to only 1.5% (78.15 ± 0.39 mV vs. 79.37 ± 0.24 mV in the control; *n *= 127, *p * < 0.05).

The infeasibility of total blockade by APV can be explained by the fact that APV is a reversible blocker that competitively binds to the glutamate-binding site of NMDAR and its affinity is close to that of glutamate; therefore, the amino acid could displace the blocker. In order to completely eliminate the effect of amino acids, we additionally applied 5,7-dichlorokynurenic acid (5,7-DCKA), an NMDAR glycine binding site blocker, at a concentration of 100 μM. Neither addition of 5,7-DCKA nor simultaneous application of APV and 5,7-DCKA affected the resting membrane potential (*n *= 79), but the combined action of 5,7-DCKA and APV prevented the effect of amino acids on the resting membrane potential (78.8 ± 0.22 mV vs. 79.37 ± 0.24 mV in control; *n* = 85, *Figure*).

**Figure:**
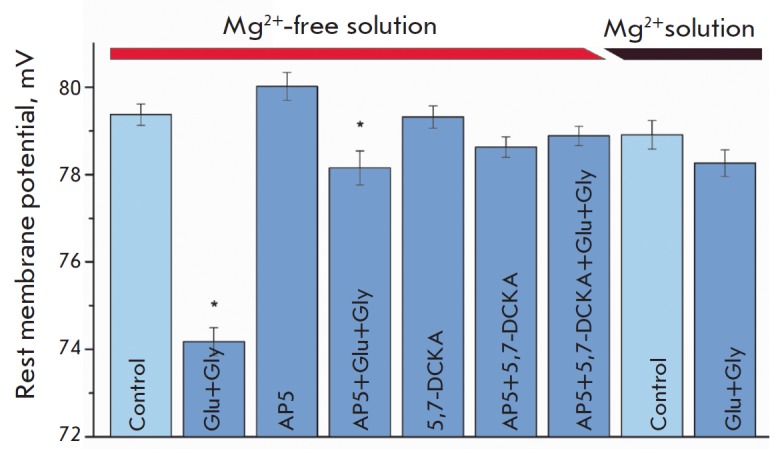
The effect of the activation of NMDA receptors on the resting membrane potential of a postsynaptic cell. The RMP value in the Mg^2+^-free solution decreased after the application glutamate (100 μM) and glycine (700 μM). Addition of NMDA receptor blockers, APV (DL-2-amino-5-phosphonopenthatoic acid, 500 μM) or 5,7-dichlorokynurenic acid (5,7-DCKA; 100 μM), or simultaneous application of these agents had no effect on RMP. Addition of glutamate and glycine against a APV background slightly reduced RMP, but the effect was completely inhibited when two blockers, APV and 5,7-dichlorokynurenic acid, were applied. In the solution containing magnesium ions, application of glutamate and glycine had no effect on the RMP of postsynaptic cell. * – statistically significant difference vs. control (*p * < 0.05)

Magnesium blockade is an alternative physiological way to block NMDAR. If the observed effects on the membrane potential are caused by the activation of these receptors, the presence of magnesium in solution should prevent the development of depolarization after NMDAR agonists are applied. Indeed, amino acids had no effect on the membrane potential in the presence of Mg^2+^. Hence, the membrane potential value in the Mg^2+^-containing solution was 78.91 ± 0.32 mV and remained unchanged (78.26 ± 0.31 mV, *n *= 105, *Figure*) after glycine and glutamate addition. These results provide grounds to infer that a population of functional NMDARs is localized on the postsynaptic membrane; their activation causes statistically significant changes in the membrane potential. This depolarization, evoked by a cation current through the receptor channel, is blocked by a selective blocker of NMDAR glutamate and glycine binding sites: it is not observed when the magnesium block is preserved.

## CONCLUSIONS

Hence, a functionally active population of NMDA receptors is present on the postsynaptic membrane of mammalian muscle fibers; their activation can change the excitability of muscle fiber and trigger a wide variety of intracellular reactions through the system of calcium-dependent secondary messengers, due to the relatively high permeability of the NMDA receptor channel to calcium ions. Given the variety of possible functions mediated by the NMDA receptor, further research into their role in the neuromuscular synapse seems to be an important and highly topical task.

## Abbreviations

NMDA: N-metyl-D-aspartate
ACh: acetylcholine
GABA: γ-aminobutyrilic acid
ATP: adenosine triphosphate
CNS: central nervous system
RMP: resting membrane potential
5,7-DCKA: 5,7-dichlorokynurenic acid
AP5: DL-2-amino-5-phosphonopenthatoic acid
